# Evaluation of the Sedative Activity of Naringenin: In Vivo Study with Pharmacokinetics and Molecular Docking Insights

**DOI:** 10.1002/open.202500114

**Published:** 2025-06-08

**Authors:** Dipu Bishwas, Md. Shimul Bhuia, Salehin Sheikh, Mohammed Alfaifi, Abdul Malik, Nikhat J. Siddiqui, Divya Jain, Mehedi Hasan Bappi, Muhammad Torequl Islam

**Affiliations:** ^1^ Department of Pharmacy Pabna University of Science and Technology Pabna 6600 Bangladesh; ^2^ Bioinformatics and Drug Innovation Laboratory Bioluster Research Center Ltd. Gopalganj 8100 Dhaka Bangladesh; ^3^ Department of Pharmacy Gopalganj Science and Technology University Gopalganj 8100 Bangladesh; ^4^ Department of Clinical Laboratory Sciences College of Applied Medical Sciences King Khalid University Abha 61421 Saudi Arabia; ^5^ Department of Pharmaceutics, College of Pharmacy King Saud University Riyadh 11451 Saudi Arabia; ^6^ Department of Internal Surgical Nursing College of Nursing King Saud University Riyadh 11451 Saudi Arabia; ^7^ Department of Microbiology School of Applied & Life Sciences Uttaranchal University Dehradun Uttarakhand 248007 India; ^8^ School of Pharmacy Jeonbuk National University Jeonju 54896 Republic of Korea; ^9^ Pharmacy Discipline Khulna University Khulna 9208 Bangladesh

**Keywords:** GABAergic pathways, in silico studies, insomnia, naringenin, pharmacokinetics

## Abstract

This research is designed to investigate the sedative effects of naringenin (NAR) and diazepam against thiopental sodium‐induced sleeping mice. NAR (5 and 10 mg kg), diazepam (DZP) (2 mg kg), flumazenil (FLN) (0.1 mg kg), and their combinations are administered intraperitoneally to mice. After 30 min, sleep is induced with intraperitoneal thiopental sodium (20 mg kg), and sleep latency and duration are measured. In silico analysis investigates the role of GABA receptors. NAR significantly reduces sleep latency and prolongs sleep duration in a dose‐dependent manner, with the combination of NAR‐10 and DZP‐2 showing a synergistic effect. The combination of the antagonist (FLN) (NAR‐10 and FLN‐0.1) indicates that latency increases and sleep duration decreases compared to NAR‐10 alone. Furthermore, in silico docking studies corroborates these results, demonstrating a significant binding affinity of NAR (−8.3 kcal mol), which is comparable to the standard ligand DZP (−8.7 kcal mol) and FLN (−7.0 kcal mol) for the GABA_A_ (6X3X) receptor, indicating a GABAergic mechanism. Pharmacokinetics and toxicity evaluations confirm NAR's potential as a safe therapeutic agent, with a high LD50 (2000 mg kg) and minimal toxicity. These findings highlight NAR's potential as a GABAergic sedative agent, requiring further research before being used clinically to treat sleep disorders.

## Graphical abstract

The study investigates the sedative potential of naringenin (NAR) through in vivo and in silico approaches. NAR significantly enhances sleep duration and reduces latency in mice, especially when combined with diazepam. Molecular docking shows strong binding to GABA_A_ receptors, suggesting a GABAergic mechanism. Pharmacokinetic and toxicity analyses support NAR's safety and drug‐likeness as a potential sedative agent.

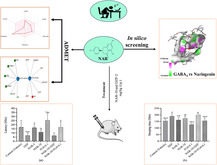

## 1 Introduction

Insomnia is defined as difficulty going to sleep, remaining asleep, or having nonrestorative sleep while having ample chance for sleep, combined with accompanying impairment of daily activities, with indications appearing for at least 4 weeks.^[^ ^1^ ^]^ In addition, insomnia is one of the most common complaints in psychiatric populations, which include adults with severe depression, and is reported in 50%–70% of those receiving primary care.^[^ ^2^ ^]^ In addition to increasing evidence that indicates, it affects not only psychological disorders but also medical issues such as hypertension, coronary heart disease, compromised immune systems, and inflammatory disease.^[^ ^3^ ^]^ According to the National Council on Aging (NCOA), roughly 30% of individuals suffer symptoms of insomnia, with 10% experiencing insomnia that impairs their daily activities. Sleep difficulties are typically related to neurodegenerative illnesses, including those originating from Alzheimer's disease, schizophrenia, bipolar disorder, and severe depression.^[^ ^4^ ^]^ Various factors, such as anxiety, depression, stress, lifestyle choices, pregnancy, age, cancer, allergies, nocturia, migraines, excessive intake of alcohol and caffeine, physical ailments, or other medical conditions, may create sleep disruptions.^[^ ^5^ ^]^

Insomnia is becoming more prevalent in contemporary culture, fueled by the rising strains of everyday life and scholastic demands.^[^ ^6^ ^]^ Gamma‐aminobutyric acid type A (GABA_A_) is a critical pharmacological target for the management of neuropsychiatric conditions, including seizures, insomnia, and anxiety.^[^ ^7^ ^]^ This receptor has been a primary target for several anesthetics.^[^ ^8^ ^]^ The GABAergic system in the mammalian brain consists of GABA‐releasing neurons and receptors that interact with GABA. The activation of GABA_A_ receptors increases chloride ion permeability, leading to neuronal inhibition^[^ ^9^ ^]^ and is extensively distributed throughout the central nervous system (CNS).^[^ ^10^ ^]^ GABA_A_ receptors have many subtypes, with the sedative and hypnotic effects mediated by the α1 and β2 subunits.^[^ ^11^ ^]^ Addressing the GABA_A_ receptor subunits may be a viable approach for developing innovative and effective sedative agents from many sources, including natural chemicals and their derivatives.

Diazepam (DZP), lorazepam, and alprazolam are benzodiazepines (BZPs) often used for the treatment of insomnia.^[^ ^12^ ^]^ However, they present numerous issues, such as confusion, dizziness, drowsiness, instability, memory deficits, sedation, muscle relaxation, anterograde amnesia, and impacts on motor functions and cognition, including impaired saccadic eye movements,^[^ ^13^ ^]^ sleep disorders,^[^ ^14^ ^]^ and increased accident risk.^[^ ^15^ ^]^ Natural ingredients may significantly contribute to eliminating these adverse effects. Natural products have garnered global study interest owing to their pharmacological properties attributed to the inclusion of fatty acids, sterols, alkaloids, flavonoids, glycosides, saponins, tannins, terpenes, and others.^[^ ^16^ ^]^ Numerous research have demonstrated the use of natural products containing diverse phytochemicals with varying pharmacological effects on the CNS or brain.^[^ ^17^ ^]^

Naringenin (NAR) (2,3‐dihydro‐5,7‐dihydroxy‐2‐(4‐hydroxyphenyl)‐4H‐1‐benzopyran‐4‐one) was identified by Power and Tutin in 1907.^[^ ^18^ ^]^ This flavonoid compound is found in several fruits, including oranges, grapefruit, and bergamot.^[^ ^19^ ^]^ It has shown several biological effects, such as anti‐inflammatory,^[^ ^20^ ^]^ antioxidant,^[^ ^21^ ^]^ hypolipidemic,^[^ ^22^ ^]^ immunomodulation, and antineoplastic.^[^ ^23^ ^]^ NAR may exhibit impacts on the central nervous system, including anxiolytic and sedative effects. Despite this potential, research on the sedative effects of NAR is limited. The research seeks to reduce the side and unfavorable effects of sedative drugs.

## 2 Results

### 2.1 In Vivo Investigations

**Figure 1:**
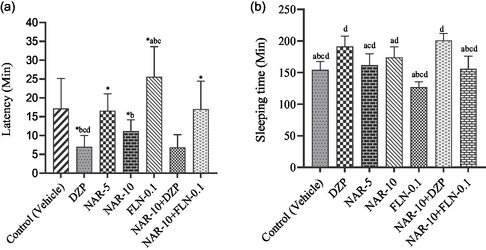
a) Latency and b) sleeping time in minutes observed in different treatment groups of animals. [Values are mean ± SEM (*n* = 7); one‐way analysis of variance followed by *t*‐students Newman‐Keuls as a post hoc test with multiple comparison; *p <* 0.05 compared to the *Control (vehicle), DZP^a^, NAR‐5^b^, NAR‐10^c^, and DZP‐2 + NAR‐10^d^; Control, vehicle (distilled water containing 0.9% NaCl and 0.5% tween 80); DZP, diazepam; FLN, flumazenil; NAR, naringenin].

In this investigation, it revealed that animals treated with the control (vehicle) exhibited delayed latency (17.14 ± 3.58 min). In contrast, the DZP group exhibited a substantial (*p* < 0.05) decrease in latency (7.00 ± 1.35 min) relative to the control group. The test substance NAR exhibited a substantial (*p* < 0.05) reduction in latency relative to the control group in a dose‐dependent manner. The average delay for the NAR‐5 and NAR‐10 groups was 16.57 ± 2.00 min and 11.14 ± 1.34 min, respectively. In the antagonist group, flumazenil (FLN)‐0.1 increased the delay (25.57 ± 3.57 min) relative to the vehicle and control groups. Conversely, the animals in the combination group (DZP‐2 + NAR‐10) demonstrated the shortest delay of 6.85 ± 1.50 min, whereas the test compound mixed with the antagonist (FLN‐0.1 + NAR‐10) group resulted in an increased latency of 17.00 ± 3.31 min, comparable to the vehicle group. The latencies in the various treatment groups are depicted visually in **Figure** **1**a.

This research indicated that the antagonist group (FLN‐0.1) of experimental mice had the shortest average sleep duration (126.85 ± 3.72 min). The DZP group exhibited a substantially longer sleeping time (191.14 ± 7.30 min) compared to the control group (*p* < 0.05). The test sample (NAR) from animal groups significantly increased sleep duration in a dose‐dependent manner compared to the control group, with an average sleep duration of 154.28 ± 5.86 min. The NAR exhibited a sleep duration of 161.71 ± 7.93 min for NAR‐5 and 174.14 ± 7.41 min for NAR‐10. In the co‐treatment group (DZP + NAR‐10), the subjects exhibited the greatest sleep duration (201.00 ± 4.74 min), whereas the test compound paired with the antagonist (FLN‐0.1 + NAR‐10) demonstrated a length of 155.71 ± 9.04 min, which was marginally greater than that of the control group. The sleep duration of various treatment groups is represented visually in Figure 1b.

### 2.2 In Silico Study

**Figure 2:**
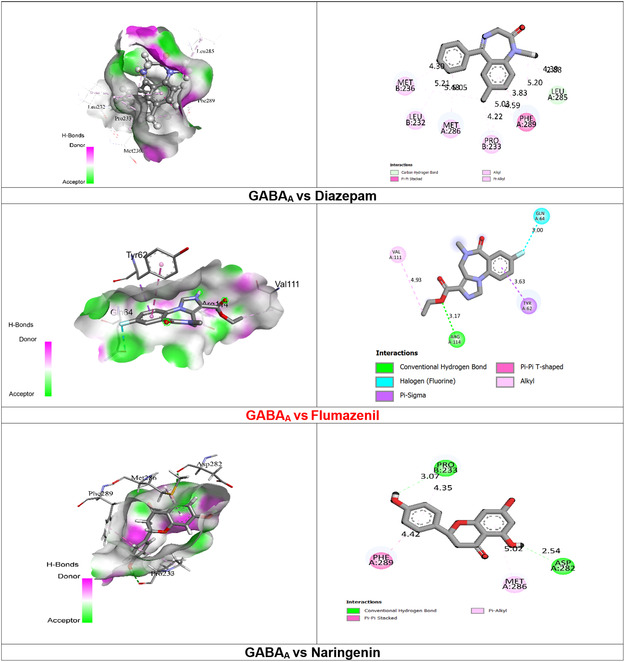
2D and 3D visualization of NAR, FLN, and DZP interactions with GABA_A_ receptors.

Our results showed that DZP, NAR, and FLN interact with GABA_A_ receptors through the formation of several kinds of bonds, including alkyl, pi‐alkyl, pi‐anion, and pi‐pi stacking bonds, as well as hydrogen bonds (HB) (both conventional and carbon HB). By forming one (HB) and multiple hydrophobic (HP) bonds with the specific amino acid (AA) residues of LEU A:285 and PHE A:289, PRO B:233, MET A:266, MET B:236, and LEU B:232, respectively, the standard ligand (DZP) showed the highest docking score of −8.7 kcal mol against the GABA_A_ receptor based on this in silico study. However, our ligand under test (NAR) demonstrated a greater binding affinity (−8.3 kcal mol). The NAR also expressed two HB with certain AA residues, such as ASP A:282 and PRO B:233, as well as two HP bonds with MET A:288 AA residues, respectively. In contrast, FLN demonstrates lower binding affinity (−7.0 kcal mol) and forms one HB, one halogen bond, and two HP bonds with specific (AA) residues, including TYR A:62 and VAL A:111. This makes NAR a more favorable candidate in this context **Table** **1** and **Figure** **2**.

**Table 1:** Molecular docking scores of NAR, FLN, and DZP against GABA_A_ receptors.

Protein‐ligand complex	Docking score [kcal mol]	AA residues	Bond types	Bond length (Å)
DZP	−8.7	LEU A:285	Carbon‐HB	4.30
		PHE A:289 PRO B:233 MET A:266 LEU B:232 MET B:236	HP	4.59 4.22 ____ 5.21 4.30
FLN	−7.0	ARG A:114	Conventional HB	3.17
		GLN A:64	Halogen	3.00
		TYR A:62 VAL A:111	HP	3.63 4.93
NAR	−8.3	PRO B:233	Conventional HB	3.07
		ASP A:282		2.54
		MET A:286	HP	5.02
		PHE A:289		4.42

DZP, diazepam; FLN, flumazenil; NAR, naringenin; HB, hydrogen bond.

#### 2.2.1 Drug‐Likeness and Pharmacokinetics

**Figure 3:**
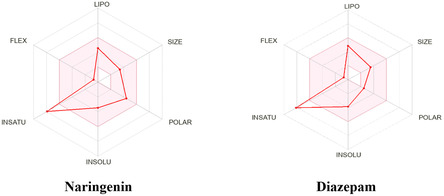
Summary of physiochemical, toxicological, and pharmacokinetics properties of selected compounds (NAR and DZP). [The colored zone is the suitable physicochemical space for oral bioavailability; SIZE, 150 g mol < MV < 500 g mol; INSOLU (insolubility), −6 < log S (ESOL) < 0; LIPO (lipophilicity), −7 < XLOGP3 < +5.0; INSATU (in saturation), 0.25 < Fraction Csp3 < 1; POLAR (polarity), 20 Å^2^ < TPSA < 130Å^2^; FLEX (flexibility), 0 < num.rotatable bonds < 9].

Molecular weight (MW), Log *P*, HB acceptor (HBA), HB donor (HBD), and molar refractivity (MR) are the major factors by which SwissADME defines the molecule as drug‐like (Feng et al. 2020). Our results showed that the standard ligand DZP has a MW below 500 Dalton, no more than 5 HBD, less than 10 HBA, a lipophilicity value not greater than 5, and MR lower than 140. On the other hand, our test ligand NAR showed a MW below 500 Dalton, no more than 5 HBD, less than 10 HBA, a lipophilicity value not greater than 5, and MR lower than 140. Other pharmacokinetic parameters of both ligands including blood–brain barrier (BBB) permeability, *P*‐gp substrate, and CYP2C19 inhibitor, along with the bioavailability score provided in **Table** **2**. A graphical presentation is also included in **Figure** **3**.

**Table 2:** Comparison of pharmacokinetic properties of naringenin and diazepam estimated by SwissADME.

Parameter	Naringenin	Diazepam
	Physicochemical properties	
MF	C_15_H_12_O_5_	C_16_H_13_ClN_2_O
MW	272.25 g mol	284.74 g mol
Num. heavy atoms	20	20
Num. arom. heavy atoms	12	12
Fraction Csp3	0.13	0.12
Num. rotatable bonds	1	1
HBA	5	2
HBD	3	0
MR	71.57	87.95
TPSA	86.99 Å^2^	32.67 Å^2^
	Solubility	
Water Solubility	Poorly soluble	Very slightly soluble
	Lipophilicity	
Log *P*_*o/w*_ (WLOGP)	2.19	2.39
	Pharmacokinetics	
GI absorption	High	High
BBB permeant	Yes	Yes
*P*‐gp substrate	Yes	No
CYP2C19 inhibitor	No	Yes
	DrugLikenss	
Lipinski	Yes; 0 violation	Yes; 0 violation
BIO Score	0.55	0.55
	Medicinal chemistry	
Synthetic accessibility	3.01	3.00

MF = molecular formula; MW = molecular weight (g/mol) (optimum = ≤ 500); Log *P* = Log *P*_*o/w*_ (MLOGP) (optimum = ≤ 5); HBA = hydrogen bond acceptor (optimum = ≤ 10); HBD = hydrogen bond donor (optimum = ≤ 5); MR = molar refractivity (optimum = ≤ 140); CYP2C19 int = CYP2C19 inhibitor; BIO Score = bioavailability score. TPSA: topological polar surface area; GI: gastrointestinal; BBB: blood–brain barrier.

#### 2.2.2 Toxicological Profile

**Figure 4:**
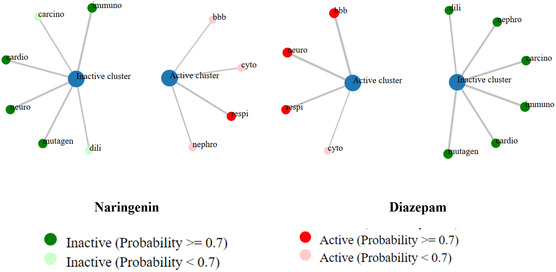
The network chart is intended to quickly illustrate the connection between the selected compound (NAR and DZP) and predicted activities. [cardio, cardiotoxicity; mutagen, mutagenicity; nephro, nephrotoxicity; dili, hepatotoxicity; carcino, carcinogenicity; immuno, immunotoxicity; neuro, neurotoxicity; bbb, blood–brain barrier; respi, respiratory toxicity].

The toxicity parameters were estimated using the ProTox 3.0. Our computational analysis estimated that the anticipated lethal dosage 50 (LD_50_) of NAR is 2000 mg kg, categorizing it as toxicity class 4. DZP had an LD_50_ of 48 mg kg body weight, signifying toxicity class 2. Furthermore, our *in silico* toxicity assessment indicates that NAR has shown no detrimental impacts on hepatotoxicity, neurotoxicity, cardiotoxicity, carcinogenicity, mutagenicity, or immunotoxicity. Nonetheless, as the prediction indicated, NAR exhibited deleterious effects on nephrotoxicity, respiratory toxicity, and cytotoxicity. Conversely, it has been shown that the referral medication DZP exhibits neurotoxicity, cytotoxicity, and respiratory toxicity. All anticipated toxicological parameters are shown in **Table** **3** and **Figure** **4**.

**Table 3:** Comparison of toxicological properties of naringenin and diazepam estimated by ProTox 3.0.

Parameters	Naringenin	Diazepam
Predicted toxicity class	4	2
Predicted LD_50_	2000 mg kg	48 mg kg
Hepatotoxicity	Inactive	Inactive
Neurotoxicity	Inactive	Active
Nephrotoxicity	Active	Inactive
Respiratory toxicity	Active	Active
Cardiotoxicity	Inactive	Inactive
Carcinogenicity	Inactive	Inactive
Immunotoxicity	Inactive	Inactive
Mutagenicity	Inactive	Inactive
Cytotoxicity	Active	Active
BBB	Active	Active

LD_50_: 50% lethal dose; BBB: blood–brain barrier.

## 3 Discussion

The sedative properties of pharmacological agents are primarily mediated through their action on the CNS, with various pathways contributing to this effect. The GABAergic system is essential for regulating neuronal excitability and facilitating sedative, anxiolytic, and muscle‐relaxant effects.^[^ ^24^ ^]^ GABA, the principal inhibitory neurotransmitter in the central nervous system, attaches to GABA receptors, resulting in an influx of chloride ions, which induces neuronal hyperpolarization and diminishes excitability.^[^ ^25^ ^]^ Numerous sedative medications amplify the effects of GABA, therefore increasing central nervous system inhibition.^[^ ^26^ ^]^ Researchers often evaluate the sedative effects of a chemical molecule by observing critical sleep indicators in animal models, including sleep onset and sleep duration.^[^ ^27^ ^]^ A substance that decreases sleep latency and extends sleep duration is often regarded as having sedative properties. These metrics provide significant insights into the compound's possible effects on the central nervous system.^[^ ^28^ ^]^

BZP, like DZP, are traditional sedatives that augment GABAergic activity by binding to the GABA_A_ receptor at a specific location, hence enhancing the receptor's affinity for GABA. This has a pronounced sedative effect, often used therapeutically for anxiety, sleep disturbances, and muscular relaxation.^[^ ^29^ ^]^ Nonetheless, these medications might induce tolerance and physical dependency in some people. For instance, DZP induces sleepiness at doses of 5 to 10 mg in novice users and may lead to tolerance in those who take it often.^[^ ^30^ ^]^ Consequently, the identification of novel, efficacious, and safer sedative pharmaceuticals is an urgent need. In this study, we used DZP as a reference medication to evaluate the sedative efficacy of our test ligand (NAR). Synergistic activity refers to the phenomenon where the collective effect of two or more substances (such as drugs, chemicals, or biological entities) exceeds the sum of their individual effects.^[^ ^31^ ^]^

To elucidate the mechanism of the sedative effect of NAR, the GABA_A_ antagonist FLN was used in our in vivo experiments. FLN is a recognized competitive antagonist at the BZP binding region of the GABA_A_ receptor.^[^ ^32^ ^]^ Its use in research is crucial for ascertaining if a sedative drug operates via the BZP‐sensitive GABAergic pathway. FLN has the capacity to inhibit the sedative and anxiolytic effects of substances that function by augmenting GABAergic transmission via the GABA_A_ receptor.[^16^, ^33^] If the sedative effects of NAR are reversed or markedly diminished by FLN treatment, this would clearly indicate that NAR's activity is, at least partially, mediated via the BZP binding site on the GABA_A_ receptor. Conversely, if FLN does not counteract the effects of NAR, it would imply that NAR could exert its sedative effects via alternate mechanisms, perhaps engaging non‐BZP sensitive sites or other neurotransmitter systems.

Our investigation revealed that DZP and NAR, when administered separately, substantially decreased sleep latency and extended sleep duration in a dose‐dependent manner relative to the control group, therefore illustrating their pronounced sedative effects in rats. Furthermore, these effects were significantly amplified when NAR was co‐administered with DZP, a recognized GABA_A_ receptor agonist. The animal cohort administered the combined therapy (DZP‐2 + NAR‐5) exhibited a substantial reduction in sleep latency to 6.85 ± 1.50 min and an increase in sleep duration (201.00 ± 4.74 min) relative to the single treatment group. The data suggest that NAR has a synergistic effect when co‐administered with DZP. Moreover, the concurrent treatment of FLN with NAR yielded significant insights. Administration of FLN in conjunction with NAR resulted in prolonged sleep latency and reduced sleep duration relative to the effects of NAR alone. This indicates that FLN may partly counteract the sedative effects of NAR, suggesting that the mechanism of action of NAR may include interaction with the BZP‐binding site on GABA_A_ receptors or a similar pathway.

**Figure 5:**
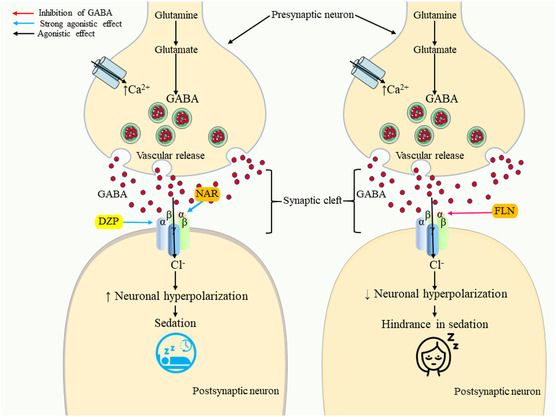
Possible sedative mechanisms of naringenin and diazepam through GABAergic pathways. [GABA, gamma‐aminobutyric acid; NAR, naringenin; DZP, diazepam; FLN, flumazenil].

Molecular docking is used to assess the binding affinity between a ligand and a certain protein.^[^ ^34^ ^]^ Our in silico analysis revealed that DZP, NAR, and FLN exhibited docking score (−8.7, −8.3, and −7.00 kcal mol, respectively) against the GABA_A_ receptor. The DZP interacts with the positive allosteric site of GABA_A_ receptors by establishing several interactions with AA residues, namely LEU A:285, PHE A:289, PRO B:233, MET B:236, and LEU B:232. Conversely, the evaluated ligand (NAR) exhibited superior binding affinity with a docking score of −8.3 kcal mol, which is nearer to the DZP. Furthermore, as a result of their interaction, numerous particular AA residues are identified, including ASP A:282, MET A:286, PRO B:233, and PHE A:289. We observed that the NAR generated two identical AA residues at the same position in GABA_A_ receptors, namely PHE A:289 and PRO B:233. Consequently, the comprehensive results indicate that NAR may exhibit sedative‐like effects akin to DZP. Furthermore, since both NAR and DZP exhibited identical binding sites on the GABA_A_ receptor, the administration of NAR during DZP therapy might enhance its concentrations in the central nervous system, thereby amplifying its efficacy. Therefore, based on the findings exhibited, NAR can be a potent drug candidate for sleeping disorders. The possible sedative mechanism of NAR in comparison to DZP and FLN are depicted in **Figure** **5**.

Drug likeness is an important parameter in drug development and discovery. It provides qualitative assessments on the probability of a chemical molecule's suitability for oral administration, predicated on its absorption and distribution capabilities inside the body.^[^ ^35^ ^]^

Lipinski's Rule of Five is often used to forecast pharmacokinetics and drug‐likeness. Lipinski's rule of five stipulates that a drug candidate must possess a MW of 500 g mol or less, a HBA count of 10 or fewer, a HBD count of 5 or fewer, and a lipophilicity (Log *P*_*o/w*_) not exceeding five.^[^ ^6^ ^]^ In our investigation, both ligands (NAR and DZP) exhibit acceptable values for all metrics. Both ligands are anticipated to exhibit outstanding pharmacokinetic characteristics and are likely to qualify as prospective medicines based on Lipinski's criterion.

Toxicological screening is an essential phase in drug development that facilitates the discovery and prioritizing of compounds with the greatest potential for safe and effective human use.^[^ ^36^ ^]^ The study's results reveal that NAR exhibited no immunotoxic, mutagenic, carcinogenic, neurotoxic, cardiotoxic, or hepatotoxic effects. Moreover, a comparison of the fatal dose (LD_50_) of our ligands with that of DZP reveals that our ligands possess a higher LD_50_, indicating they are less toxic than DZP when supplied in equivalent quantities. Nonetheless, it exhibited detrimental effects concerning renal impairment, negative impacts on the respiratory system, and induced cytotoxicity. The importance of the degree may be reduced since our computer analysis did not reveal any concerning probability.

Collectively, our test results indicated that NAR had a pronounced sedative effect in experimental mice by extending sleep duration and decreasing sleep onset. The computer analysis identified the fundamental mechanisms responsible for the sedative effects, maybe by exploring the interaction between NAR and different AA residues of GABA_A_ receptors.

## 4 Conclusion

This study examined the possible sedative effects of NAR in mice driven to sleep using TS. In contrast to the control group, the in vivo tests showed that NAR accelerated the initiation of sleep and increased the duration of sleep. Additionally, when combined with DZP, NAR had synergistic effects that improved sleep duration and initiation. The in silico investigation indicated that NAR effectively interacted with the α1 and β2 subunits of the GABA_A_ receptors, achieving docking scores of −8.3 kcal mol, which is comparable to DZP and FLN's score of −8.7 and −7.0 kcal mol, respectively. Pharmacokinetic studies further demonstrated that NAR may be a safer substitute due to its high GI absorption and reduced toxicity (LD_50_ 2000 mg kg^−1^) in comparison to DZP (LD_50_ 48 mg kg). Even while FLN counteracted the effects of NAR, its partial counteraction raises the possibility of combined treatment. Nonetheless, we recommend more preclinical studies to look at the regulation of wakefulness and sleep as well as the true underlying reasons of sedatives triggered on by NAR. The results of this study will help guide the clinical development of new medications intended to treat this extremely common ailment in a secure and effective way.

## 5 Experimental Section

5.1

5.1.1

##### Chemicals and Reagents

Square and Opsonin Pharmaceuticals Ltd., Bangladesh, supplied DZP, flumazenil (FLN), and water for injection (WFI) and thiopental sodium (TS), respectively. Sigma–Aldrich (USA) supplied NAR (CAS No: 67604‐48‐2, purity: 98%), while Merck (Mumbai, India) supplied tween 80.

##### Selection and Preparation of Test and Control Groups

Two (5 and 10 mg kg) doses of the test substance (NAR) were chosen after a thorough literature assessment.^[^ ^37^ ^]^ First, we adjusted with WFI to prepare a stock solution at a concentration of 10 mg kg (higher dose). We diluted the stock solution to get the lower dose, which was 5 mg kg. At dose of 2 mg kg, the standard drug DZP solution was made by fully combining it with WFI. Ultimately, the antagonist formulation was made by carefully combining the WFI and FLN solutions to a concentration of 0.1 mg kg. To understand the combined action of NAR and DZP, a maximum dose of each drug was utilized as part of combination therapy.

##### Experimental Animals

*Swiss* *albino* mice, with an average body weight of 21 to 25 g, were acquired from the animal house of Jahangirnagar University in Savar, Bangladesh. Prior to the experiment, the animals were housed at the pharmacy department's pharmacology lab at Gopalganj Science and Technology University (GSTU), Gopalganj 8100, Bangladesh, at a consistent temperature of 28 ± 1 °C (relative humidity: 65%) and under controlled lighting (12‐h dark/light cycle). Regular quantities of food and water were always available to the animals without charge. In order to look for any potential poststudy fatalities, the mice were observed for 17 h throughout the current trial, which ran from 8:00 a.m. to 3:00 p.m. The GSTU Animal Ethics Committee gave its approval to this investigation (#GSTU‐pharmacy‐20PHR001).

##### In Vivo Protocol

Forty‐nine animals in all were randomly divided into seven groups, each with seven animals, following a 4‐day adjustment period. The test sample (NAR‐5, 10), standard medication (DZP), (DZP‐2 + NAR‐10), antagonist (FLN) (FLN‐0.1 + NAR‐10) and vehicle (control) were then administered intraperitoneally (i.p.). Each animal received 20 mg kg b.w. of TS (i.p.) to induce sleep after a 30‐min treatment period, and it was then put in an observation room (like a plastic cage). Following TS injection, the righting reflex was lost, and the latent time was noted. Through physical observation, the length of time an individual slept or the time between losing and returning the reaction was also meticulously recorded.

##### Statistical Analysis

The sedative effectiveness results are shown as the mean and standard error of the mean (SEM). The Graph Pad Prism (version 6.0) is a statistical software program used to calculate the statistical significance of fluctuations, with a confidence level of 95%. *p* Values below 0.05 are thought to be significant.

##### In Silico Analysis: Selection of GABA_A_ Macromolecule and Preparation

We have selected the α1 and β2 subunits of the GABA_A_ receptor, which mediate sedation, based on prior research.^[^ ^38^ ^]^ The RCSB Protein Data Bank provided the 3D structures of the GABA_A_ receptor (PDB ID: 6X3X) subunits‐α1 in the B chain and β2 in the A chain.^[^ ^39^ ^]^ Procedures intended to remove docking interference after collection were used to improve the receptors. The macromolecules were improved by eliminating extraneous heteroatoms, water molecules, and AA residues using the program PyMOL version 1.7.4.5.^[^ ^40^ ^]^ The Swiss‐PDB Viewer software was then used to adjust the GROMOS96 43 B1 force field, which decreased the energy consumption of the protein structures.^[^ ^41^ ^]^

##### Collection and Preparation of Ligands

**Figure 6:**
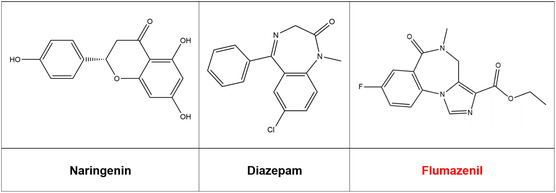
Chemical structures of naringenin, flumazenil, and diazepam.

The “sdf” file format was used to retrieve the chemical structures for the standard medication DZP (PubChem ID: 3016), FLN (PubChem ID: 3373), and NAR (PubChem ID: 439246) from the chemical database PubChem. Chem3D Pro21.0 software used the Allinger's force field (MM2) technique to reduce the ligands’ energy.^[^ ^42^ ^]^ **Figure** **6** shows the 2D conformation of NAR, DZP, and FLN.

##### Docking Protocol and Nonbond Interactions

Molecular docking is a common computational tool used in pharmaceutical investigations for drug development.^[^ ^43^ ^]^ This method assesses the pharmacodynamic characteristics of drugs by analyzing and correlating molecules to certain binding sites using the PyRx v0.8 software.^[41a]^ The outcome of the docking process indicates the strength of the interaction between a ligand and the active site of a particular protein. In order to speed up the docking process, the size of the grid box dimensions along the X, Y, and Z axes were maximized.^[^ ^42^ ^]^ The computation was then executed 200 times. The PDB format of the ligand‐protein complex was acquired in order to obtain the ligand in PDBQT format.^[^ ^44^ ^]^ Previous research found that the binding affinity value of the ligand was expressed as a negative value in kcal mol.^[^ ^45^ ^]^ Furthermore, an investigation of the active binding areas of the proteins is undertaken by the application of BIOVIA Discovery Studio v21.1.0, which permits the identification of non‐bond interactions within the ligand‐protein complexes.^[40a]^

##### Prediction of Pharmacokinetics and Drug‐Likeness

The ADMET characteristics, which evaluate the body's response to medications over time, are the foundation of statistical pharmacokinetics investigations. The in silico approach aids in the conversion of a material into a therapeutically useful medication by analyzing the early pharmacokinetic characteristics of a chemical. In research and development, the qualitative metric known as “drug‐likeness” measures how closely a chemical resembles a medication under specific conditions.^[^ ^46^ ^]^ The SwissADME database[^16^, ^25^, ^30^] was used to evaluate ERI and the standard for drug‐likeness and ADMET functions.

##### Toxicity Prediction

A crucial step in the drug development process, toxicity prediction helps identify and rank the compounds having the best chance of being used safely and effectively by people. Additionally, this method reduces the possibility of costly drug development failures later on.^[^ ^47^ ^]^ Any particular drug's toxicity parameters may be predicted using the ProTox 3.0 web server.^[^ ^48^ ^]^ The canonical SMILES acquired from PubChem were sent to the ProTox 3.0 website (http://tox.charite.de/protox‐3) for evaluation of the toxicity parameters. The server then identified the chemical's hazard class and a number of other properties. **Table** **4** documents the analysis and documentation of the selected chemicals’ various toxicity parameters.

**Table 4:** Different treatments and their doses were investigated in animals.

Treatment group	Description	Dose (mg/kg)	Target receptor
Control (vehicle)	Distilled water containing 0.9% NaCl and 0.5% tween 80	10 ml kg	–
DZP	Standard: diazepam (agonist) (i.p)	2	GABA_A_
FLN‐0.1	Antagonist	0.1	–
NAR‐5	Lower dose	5	Under investigations
NAR‐10	Higher dose	10	–
DZP‐2 + NAR‐10	Standard + test combination	2 + 10	–
FLN‐0.1 + NAR‐10	Antagonist + Test combination	0.1 + 10	–

Control (vehicle): distilled water containing 0.9% NaCl and 0.5% tween 80; DZP: diazepam (dose: 2 mg kg); NAR: naringenin (dose: 5 and 10 mg kg); FLN: flumazenil (dose: 0.1 mg kg); all treatments provided through (i.p) route.

## Conflict of Interest

The authors declare no conflict of interest.

## Author Contributions

All authors made a significant contribution to the work reported, whether that is in the conception, study design, execution, acquisition of data, analysis, and interpretation, or in all these areas, that is, revising or critically reviewing the article; giving final approval of the version to be published; agreeing on the journal to which the article has been submitted; and confirming to be accountable for all aspects of the work. All authors have read and agreed to the published version of the manuscript. **Dipu Bishwas**: conceptualization (equal) and writing. **Md. Shimul Bhuia**: conceptualization (equal), writing and editing. **Salehin Sheikh**: formal analysis (equal); software (supporting); and supervision (equal). **Mohammed Alfaifi**: supervision (lead) and validation (equal). **Abdul Malik**: formal analysis (lead) and funding acquisition (supporting). **Nikhat J. Siddiqui**: validation (lead) and visualization (supporting). **Divya Jain**: methodology (lead) and visualization (supporting). **Mehedi Hasan Bappi**: conceptualization (lead); data curation (lead); project administration (lead); supervision (lead); and visualization (lead). All authors have read and agreed to the published version of the manuscript.

## Data Availability

The data that support the findings of this study are available from the corresponding author upon reasonable request.
